# More than Fluid: AI-Derived Pleural Effusion Volume as a Marker of Cardiovascular Congestion in Transcatheter Aortic Valve Implantation

**DOI:** 10.3390/medsci14040472

**Published:** 2026-08-11

**Authors:** Nikolaos Schörghofer, Nikolaus Clodi, Gretha Hecke, Matthias Hammerer, Alexander Kupferthaler, Bernhard Scharinger, Uta C. Hoppe, Klaus Hergan, Elke Boxhammer, Christoph Knapitsch

**Affiliations:** 1 Department of Radiology, Paracelsus Medical University of Salzburg, 5020 Salzburg, Austriac.knapitsch@salk.at (C.K.); 2 Division of Cardiology, Department of Internal Medicine II, Paracelsus Medical University of Salzburg, Müllner Hauptstraße 48, 5020 Salzburg, Austria

**Keywords:** transcatheter aortic valve implantation, pleural effusion, artificial intelligence, computed tomography, cardiovascular congestion, right ventricular function

## Abstract

**Background/Objectives:** Pleural effusions are frequently encountered on pre-procedural computed tomography (CT) scans in patients undergoing transcatheter aortic valve implantation (TAVI). While often regarded as a marker of congestion and advanced cardiovascular disease, their clinical and prognostic significance in contemporary TAVI populations remains poorly understood. This study investigated the relationship between AI-derived pleural effusion volume, cardiovascular dysfunction, and long-term mortality after TAVI. **Methods:** Consecutive patients undergoing transfemoral TAVI between 2016 and 2022 who had available pre-procedural CT imaging were retrospectively included. Pleural effusion volume was quantified using an artificial intelligence-based segmentation workflow and analyzed as categorical, continuous, log-transformed, and threshold-based variables. Associations with clinical and echocardiographic characteristics were evaluated using Spearman correlation analyses. Long-term mortality was assessed using Kaplan–Meier analysis, Cox proportional hazards regression, and restricted cubic spline models. **Results:** A total of 470 patients were included (median age 82 years, 50.9% male). Pleural effusion was present in 124 patients (26.38%), including 73 (15.53%) with small, 26 (5.53%) with moderate, and 25 (5.32%) with larger effusions. Increasing pleural effusion volume was associated with atrial fibrillation (AF) (rho = 0.189, *p* < 0.001), higher systolic pulmonary artery pressure (rho = 0.224, *p* < 0.001), lower tricuspid annular plane systolic excursion (rho = −0.236, *p* < 0.001), impaired right ventricular–pulmonary arterial coupling (rho = −0.267, *p* < 0.001), lower stroke volume index (rho = −0.229, *p* < 0.001), and reduced left ventricular ejection fraction (rho = −0.221, *p* < 0.001). Despite these associations, pleural effusion volume was not associated with long-term mortality in univariable analyses, multivariable Cox regression models, or restricted cubic spline analyses. In the fully adjusted model, log-transformed pleural effusion volume was not independently associated with mortality (HR 1.00, 95% CI 0.93–1.08; *p* = 0.976). **Conclusions:** AI-derived pleural effusion volume is associated with markers of cardiovascular congestion and adverse hemodynamic remodeling in patients undergoing TAVI. However, pleural effusion burden does not independently predict long-term mortality, suggesting that its value lies primarily in phenotyping cardiovascular disease severity rather than risk stratification.

## 1. Introduction

Every transcatheter aortic valve implantation (TAVI) planning computed tomography (CT) contains findings that never make it into the final report. While considerable attention is devoted to annular dimensions, vascular access, and valve sizing [1], numerous additional imaging features remain unnoticed, unmeasured, or simply dismissed as incidental observations. Pleural effusion is among the most common of these findings, which is readily visible and frequently encountered.

Pleural effusions rarely occur in isolation. In cardiovascular medicine, they are commonly regarded as manifestations of elevated filling pressures, pulmonary hypertension, right-heart dysfunction, renal impairment, or systemic congestion [2,3,4]. In other words, they may represent the cumulative consequence of several pathophysiological processes known to influence outcomes in patients with advanced heart disease [5]. Yet despite their biological plausibility, pleural effusions are usually reduced to a binary observation: present or absent. Whether they merely reflect disease severity or identify a clinically relevant phenotype remains largely unknown.

The question may be particularly relevant in patients undergoing TAVI. Severe aortic stenosis (AS) is increasingly recognized not simply as a disease of the valve but as a complex syndrome of myocardial remodeling, pulmonary vascular dysfunction, atrial disease, congestion, frailty, and biological aging [6,7,8]. Within this framework, pleural effusion may represent far more than an incidental imaging finding. It may serve as an integrative marker of cardiovascular vulnerability, reflecting the cumulative burden of cardiac and extracardiac dysfunction that develops over years of progressive disease [3].

Risk stratification in patients undergoing TAVI is inherently multidimensional. Beyond conventional surgical risk scores, adverse outcomes have been associated with clinical factors including frailty, comorbidity burden, renal dysfunction, and impaired functional status [9]. Imaging-derived markers provide additional information on the extent of cardiac damage, including left ventricular dysfunction, pulmonary hypertension, right ventricular dysfunction, and impaired right ventricular–pulmonary arterial coupling [6]. Moreover, routinely acquired pre-procedural CT imaging offers an opportunity to assess additional markers of biological vulnerability, including body composition and sarcopenia [10]. Together, these parameters illustrate that prognosis after TAVI is determined not solely by procedural success or valvular hemodynamics, but by the cumulative burden of cardiac and extracardiac disease [8].

Until recently, however, studying pleural effusions in this context was challenging. Most available data rely on chest radiography, echocardiography, or qualitative CT assessment [11,12,13], approaches that provide only limited information regarding the true extent of fluid accumulation. Advances in artificial intelligence (AI) have fundamentally changed this landscape. Automated image segmentation now enables rapid and reproducible volumetric quantification of pleural effusions from routinely acquired CT datasets, transforming a previously neglected observation into a measurable imaging biomarker [14,15].

The clinical relevance of pleural effusions in patients undergoing TAVI therefore remains uncertain. While their presence intuitively suggests advanced cardiovascular disease, it is unclear whether pleural effusion volume represents a meaningful marker of cardiac dysfunction or merely accompanies the complex remodeling processes that characterize severe AS, especially in aging populations. The ability to quantify pleural effusion burden objectively using artificial intelligence now provides an opportunity to address this question. Accordingly, we evaluated the association between AI-derived pleural effusion volume, markers of cardiovascular dysfunction, and long-term mortality in a contemporary TAVI population.

## 2. Materials and Methods

### 2.1. Study Design and Population

This retrospective single-center observational cohort study was conducted at Paracelsus Medical University Hospital Salzburg, Austria, and included consecutive patients who underwent TAVI for severe symptomatic AS between January 2016 and June 2022.

All patients underwent comprehensive pre-procedural clinical, laboratory, echocardiographic, and CT assessment according to institutional standards and contemporary guideline recommendations. Clinical characteristics, imaging findings, procedural data, and follow-up information were obtained from institutional databases and electronic medical records.

A total of 585 consecutive patients were initially screened. Patients without available pre-procedural CT imaging and those with insufficient image quality for reliable automated pleural effusion quantification were excluded. The final study population comprised 470 patients.

The study was conducted in accordance with the Declaration of Helsinki and was approved by the local ethics committee, Ethikkommission für das Bundesland Salzburg (EK-Nr. 1082/2024), on 14 August 2024.

### 2.2. CT Acquisition

All patients underwent routine electrocardiography-gated contrast-enhanced computed tomography angiography (CTA) for TAVI planning. Imaging was performed using either a 256-slice CT scanner (Revolution, General Electric Healthcare, Chicago, IL, USA) or a 128-slice dual-source CT scanner (Somatom Definition AS+, Siemens Healthcare, Erlangen, Germany).

Acquisition protocols were adapted according to patient characteristics using automatic tube current modulation and individualized tube voltage settings. Following administration of iodinated contrast medium, image acquisition extended from the thoracic inlet to the proximal femoral arteries. Images were reconstructed using standard soft-tissue kernels and transferred for subsequent AI-based analysis.

### 2.3. AI-Based Pleural Effusion Quantification

Pleural effusion volume was quantified using an artificial intelligence-based image analysis workflow applied to routine pre-procedural CT datasets.

Automated segmentation was performed using TotalSegmentator (version 2.12.0; University Hospital Basel, Basel, Switzerland), a deep learning-based framework for comprehensive anatomical segmentation of CT images [16]. The AI-generated segmentations of the pleural space were reviewed for quality assurance and subsequently used for volumetric quantification of pleural fluid. Pleural effusion volume was calculated in milliliters (mL) and served as the primary imaging variable throughout the study.

**Figure 1:**
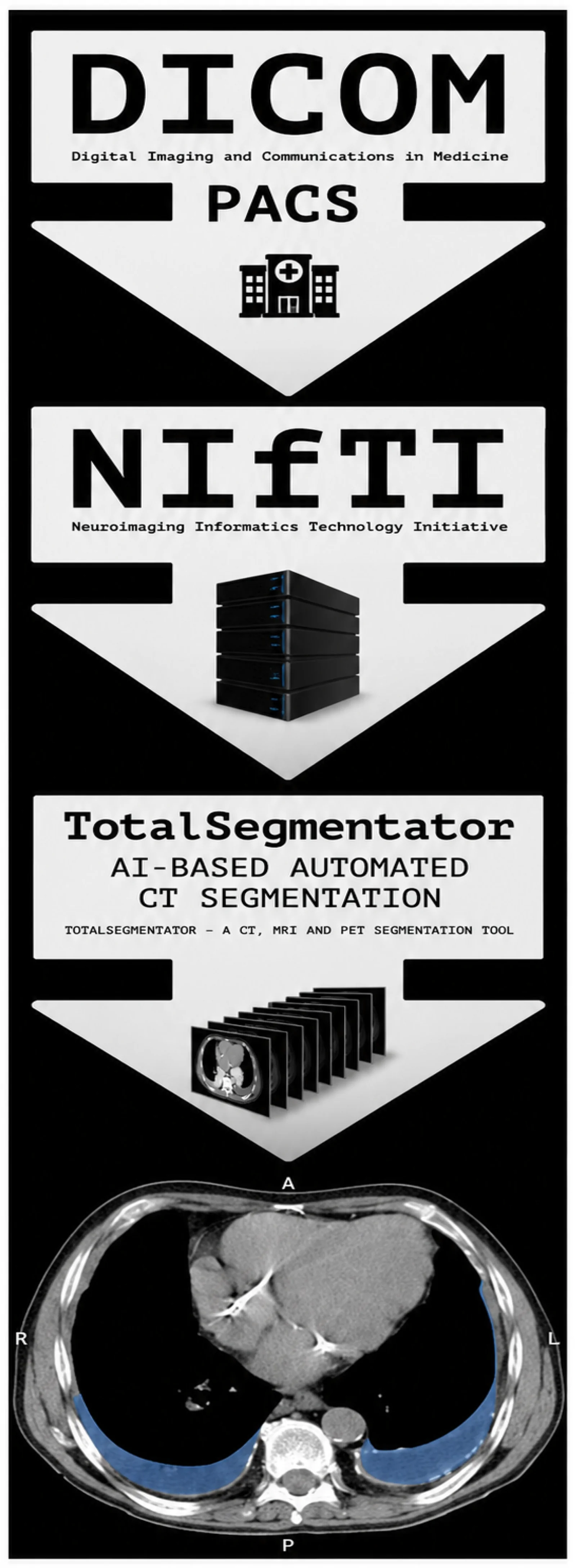
Workflow for automated CT-based pleural effusion segmentation and volumetric assessment. Contrast-enhanced chest CT scans were exported from the institutional PACS in DICOM format, converted to NIfTI, and processed using the TotalSegmentator framework for automated segmentation. AI-generated segmentation masks enabled fully automated identification and volumetric quantification of pleural effusions from routine clinical CT examinations.

A schematic overview of the segmentation and quantification workflow is provided in Figure 1.

### 2.4. Definition of Pleural Effusion Categories

For descriptive analyses, patients were categorized according to AI-derived pleural effusion volume into four groups: no pleural effusion (0 mL), small pleural effusion (>0–100 mL), moderate pleural effusion (100–500 mL), and larger pleural effusion (>500 mL).

In addition, pleural effusion volume was analyzed as a continuous variable, as a log-transformed continuous variable [log(pleural effusion volume + 1)], and according to predefined thresholds of ≥100 mL and ≥500 mL. Because of the markedly right-skewed distribution of pleural effusion volume, logarithmic transformation was used for regression and spline analyses.

### 2.5. Echocardiographic Assessment

Comprehensive transthoracic echocardiography was performed as part of the routine pre-procedural evaluation before TAVI using dedicated cardiovascular ultrasound systems (iE33 and EPIQ 5, Philips Healthcare, Hamburg, Germany).

Assessment included characterization of AS severity, left ventricular systolic function, transvalvular hemodynamics, pulmonary pressures, and right ventricular performance. Left ventricular ejection fraction (LVEF) was quantified using the biplane Simpson method whenever image quality permitted. Mean transvalvular gradients were obtained using continuous-wave Doppler echocardiography according to current recommendations.

Stroke volume index (SVi) was derived from left ventricular outflow tract measurements and Doppler velocity–time integrals and indexed to body surface area. Right ventricular systolic function was assessed by tricuspid annular plane systolic excursion (TAPSE) obtained from M-mode recordings of the lateral tricuspid annulus.

Systolic pulmonary artery pressure (sPAP) was estimated from peak tricuspid regurgitation velocity using the modified Bernoulli equation with the addition of estimated right atrial pressure derived from inferior vena cava dimensions and respiratory variation. Right ventricular–pulmonary arterial coupling was assessed using the TAPSE/sPAP ratio.

All measurements were obtained according to contemporary recommendations of the European Association of Cardiovascular Imaging (EACVI) and current European valvular heart disease guidelines [17,18,19,20].

### 2.6. TAVI Procedure

All procedures were performed via transfemoral access using contemporary self-expanding transcatheter heart valve systems, including CoreValve Evolut R and Evolut Pro (Medtronic Inc., Minneapolis, MN, USA). Procedures were performed by experienced structural heart teams according to institutional practice standards.

### 2.7. Outcome Assessment

The primary endpoint of the study was all-cause mortality following TAVI.

Vital status was determined through review of hospital records, structured telephone follow-up, communication with treating physicians, and verification through official mortality registries. Follow-up duration was calculated from the date of TAVI to death or last available clinical contact.

### 2.8. Statistical Analysis

Continuous variables are presented as median and interquartile range (IQR), whereas categorical variables are reported as counts and percentages. Comparisons across pleural effusion categories were performed using the Kruskal–Wallis test for continuous variables and the χ^2^ test or Fisher’s exact test, as appropriate, for categorical variables.

To investigate associations between pleural effusion volume and baseline characteristics, Spearman rank correlation analyses were performed for continuous variables. For binary variables, Spearman correlation coefficients were calculated using dichotomous coding (0/1).

Overall survival was assessed using Kaplan–Meier analysis and compared using the log-rank test. Survival analyses were performed both according to predefined pleural effusion categories (no effusion, small effusion, moderate effusion, and larger effusion) and according to the presence or absence of pleural effusion.

The association between pleural effusion volume and long-term mortality was evaluated using Cox proportional hazards regression models. Pleural effusion was analyzed as a binary variable (presence vs. absence), as a continuous variable per 100 mL increase, as a log-transformed continuous variable [log(pleural effusion volume + 1)], and according to predefined thresholds (≥100 mL and ≥500 mL). Results are reported as hazard ratios (HRs) with 95% confidence intervals (CIs).

Two multivariable adjustment models were constructed. Model 1 was adjusted for age, sex, atrial fibrillation (AF), and serum creatinine. Model 2 was additionally adjusted for sPAP, TAPSE, LVEF, and stroke volume index (SVi).

Potential nonlinear associations between pleural effusion volume and mortality were explored using restricted cubic spline Cox regression models with four knots. Both unadjusted and adjusted spline models were evaluated, and Wald χ^2^ statistics were used to assess overall and nonlinear effects.

The proportional hazards assumption was assessed using Schoenfeld residuals. All statistical analyses were performed using R version 4.2.3 (R Foundation for Statistical Computing, Vienna, Austria). All tests were two-sided, and a *p*-value < 0.05 was considered statistically significant.

## 3. Results

### 3.1. Distribution of Pleural Effusion Volume

Among the 470 patients included in the final analysis, 346 (73.6%) showed no evidence of pleural effusion on pre-procedural CT imaging. Small pleural effusions (>0 to <100 mL) were present in 73 patients (15.5%), moderate pleural effusions (100 to <500 mL) in 26 patients (5.5%), and larger pleural effusions (≥500 mL) in 25 patients (5.3%) (Table 1). Overall, pleural effusion was detected in 124 patients (26.4%).

### 3.2. Baseline Characteristics According to Pleural Effusion Volume

Baseline characteristics stratified according to pleural effusion burden are summarized in Table 2. Patients with larger pleural effusions exhibited a clinical profile consistent with more advanced cardiovascular disease and congestion. The prevalence of AF increased progressively across pleural effusion categories, ranging from 32.1% in patients without pleural effusion to 68.0% among those with larger pleural effusions (*p* < 0.001). Furthermore, patients with greater pleural effusion volumes demonstrated lower hemoglobin concentrations and higher serum creatinine levels.

Pronounced differences were also observed in echocardiographic parameters. Increasing pleural effusion burden was associated with lower left ventricular ejection fraction, higher systolic pulmonary artery pressure, lower TAPSE values, reduced TAPSE/sPAP ratios, and lower stroke volume index (all *p* < 0.001). In contrast, age, body surface area, diabetes mellitus, chronic obstructive pulmonary disease, previous stroke, history of malignancy, and transvalvular gradients did not differ significantly between groups.

### 3.3. Correlations Between Pleural Effusion Volume and Baseline Characteristics

Correlation analyses confirmed significant associations between pleural effusion volume and several markers of cardiac dysfunction and congestion (Table 3). The largest correlation coefficients were observed for TAPSE/sPAP (rho = −0.267, *p* < 0.001), TAPSE (rho = −0.236, *p* < 0.001), stroke volume index (rho = −0.229, *p* < 0.001), systolic pulmonary artery pressure (rho = 0.224, *p* < 0.001), and left ventricular ejection fraction (rho = −0.221, *p* < 0.001). Pleural effusion volume was also positively associated with AF (rho = 0.189, *p* < 0.001) and serum creatinine (rho = 0.186, *p* < 0.001).

Overall, the observed correlation coefficients were modest in magnitude, suggesting that pleural effusion volume reflects multiple interconnected aspects of cardiovascular remodeling rather than a single dominant pathophysiological process.

### 3.4. Pleural Effusion Volume and Long-Term Mortality

**Figure 2:**
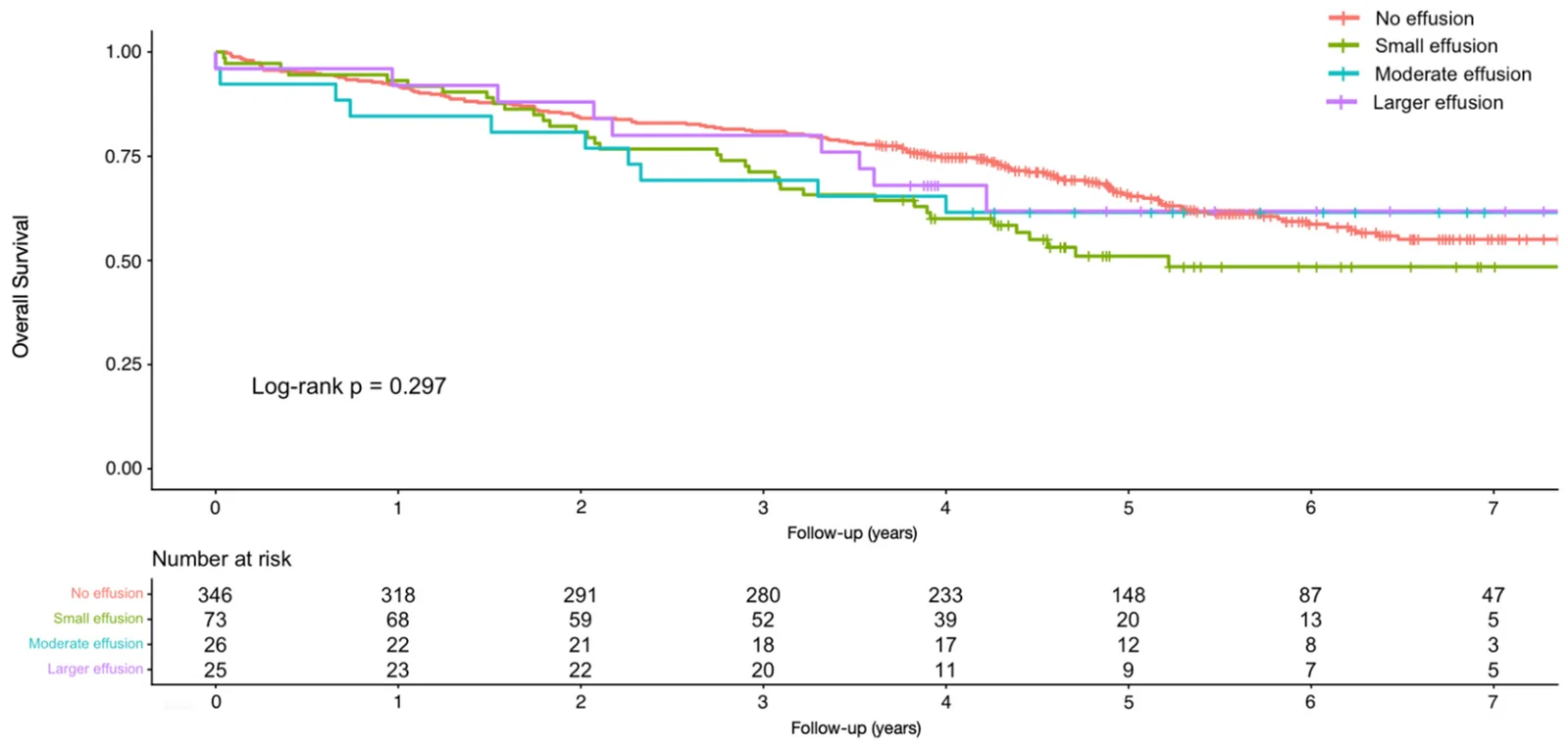
Kaplan–Meier survival curves according to pleural effusion burden. Long-term all-cause mortality stratified by pleural effusion volume categories (no effusion, small effusion, moderate effusion, and larger effusion). Pleural effusion burden was not associated with long-term mortality (log-rank *p* = 0.297).

**Figure 3:**
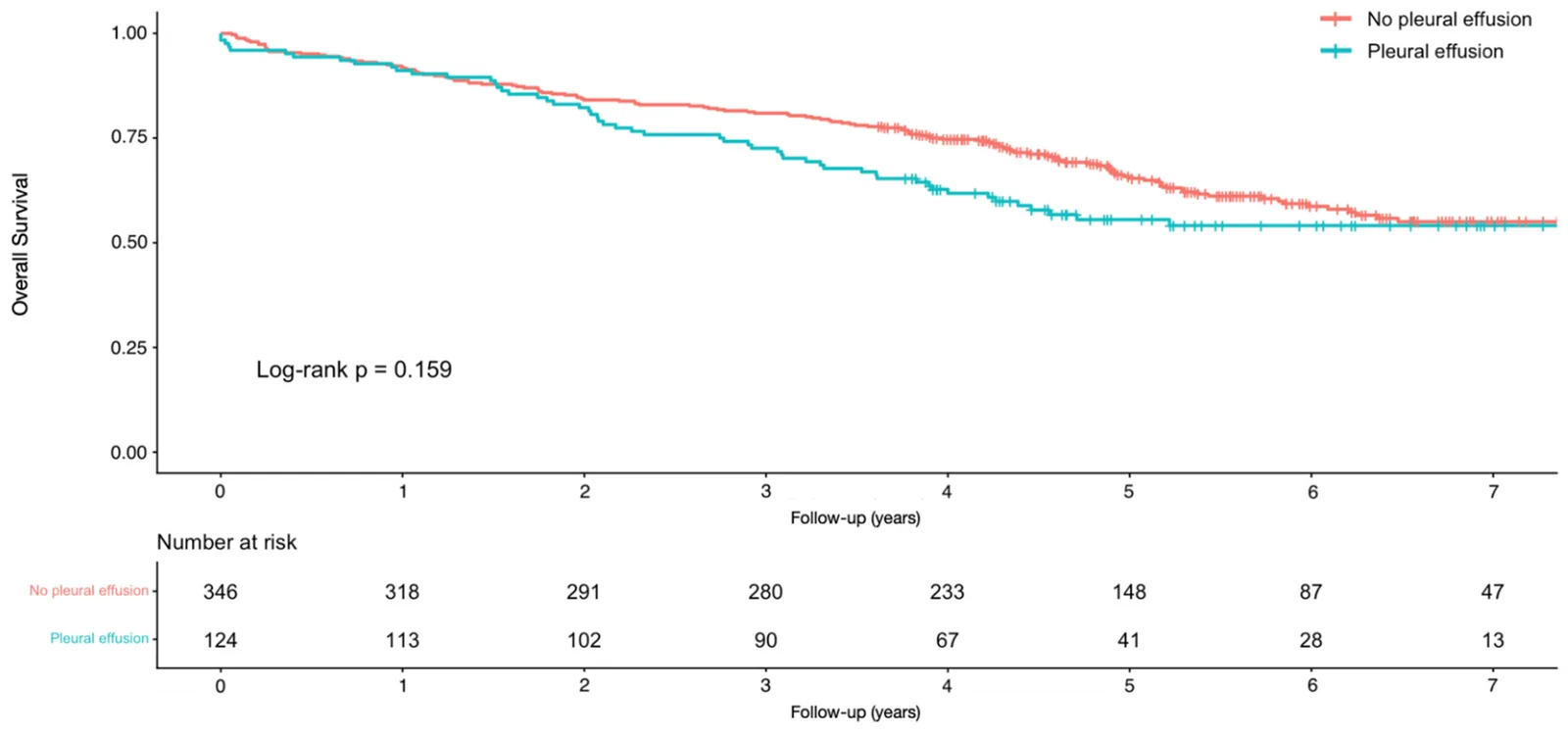
Kaplan–Meier survival curves according to pleural effusion presence. Long-term all-cause mortality in patients with and without pleural effusion on pre-procedural CT imaging. No significant difference in survival was observed between groups (log-rank *p* = 0.159).

During a median follow-up of approximately 7 years, Kaplan–Meier analyses demonstrated no significant differences in overall survival across pleural effusion categories (log-rank *p* = 0.297; Figure 2) or according to the presence or absence of pleural effusion (log-rank *p* = 0.159; Figure 3). Although patients with pleural effusion tended to exhibit numerically lower survival rates during follow-up, these differences did not reach statistical significance.

Consistent with the Kaplan–Meier analyses, univariable Cox regression models demonstrated no significant association between pleural effusion volume and long-term mortality, irrespective of the analytical approach used. Neither the presence of pleural effusion (HR 1.25, 95% CI 0.91–1.72, *p* = 0.160), pleural effusion volume per 100 mL increase (HR 1.02, 95% CI 0.97–1.07, *p* = 0.482), log-transformed pleural effusion volume (HR 1.02, 95% CI 0.95–1.10, *p* = 0.515), nor predefined thresholds of ≥100 mL or ≥500 mL were associated with mortality (Table 4).

These findings remained unchanged after multivariable adjustment. In both Model 1 and Model 2, none of the investigated pleural effusion metrics independently predicted long-term mortality. For the fully adjusted Model 2, the hazard ratio for log-transformed pleural effusion volume was 1.00 (95% CI 0.93–1.08; *p* = 0.976). Similar results were observed for all categorical and continuous pleural effusion measures (Table 5).

To further explore potential nonlinear associations, restricted cubic spline analyses were performed. Neither unadjusted nor adjusted spline models demonstrated evidence of a significant overall association or nonlinear relationship between pleural effusion volume and long-term mortality. Results were consistent across all spline models, including clinically adjusted and hemodynamically adjusted analyses (Supplementary Table S1).

## 4. Discussion

### 4.1. Principal Findings

In this retrospective single-center study of 470 patients undergoing TAVI, pleural effusion was identified in approximately one-quarter of patients on routine pre-procedural CT imaging. Increasing pleural effusion burden demonstrated consistent associations with markers of cardiovascular dysfunction and congestion, including AF, impaired right ventricular function, elevated pulmonary artery pressures, lower stroke volume index, and reduced left ventricular systolic function. These findings suggest that pleural effusions reflect the cumulative hemodynamic consequences of advanced cardiovascular disease rather than an isolated imaging abnormality. However, despite these associations, pleural effusion volume was not independently associated with long-term mortality across a broad range of analytical approaches, including categorical, continuous, multivariable, and spline-based analyses. Collectively, our findings indicate that pleural effusion primarily represents a marker of cardiovascular remodeling and congestion rather than an independent prognostic determinant in patients undergoing TAVI.

### 4.2. Pleural Effusion as a Marker of Hemodynamic Burden and Cardiovascular Remodeling

Rather than representing an isolated imaging finding, pleural effusion burden was associated with a constellation of variables reflecting chronic hemodynamic stress and cardiovascular remodeling in patients receiving TAVI. Patients with larger pleural effusions exhibited higher pulmonary artery pressures, impaired right ventricular systolic function, reduced right ventricular–pulmonary arterial coupling, lower stroke volume index, and a higher prevalence of AF. Although these correlations were modest in magnitude, the largest coefficients were observed for TAPSE/sPAP, TAPSE, and sPAP, suggesting that pleural effusion burden may partly reflect right-sided cardiac dysfunction and hemodynamic impairment.

These findings are physiologically plausible. The development of pleural effusions is closely related to elevations in pulmonary capillary hydrostatic pressure and impaired lymphatic drainage, both of which may arise in the setting of chronic left-sided pressure overload and pulmonary vascular remodeling [21,22]. In severe AS, longstanding pressure overload initiates a cascade of structural and functional changes that extends far beyond the stenotic valve itself. Progressive left ventricular remodeling, left atrial dysfunction, secondary pulmonary hypertension, and eventual right ventricular impairment are increasingly recognized as integral components of the disease process rather than isolated downstream consequences [6,23,24]. Within this framework, pleural fluid accumulation may be viewed as a clinically visible manifestation of advanced hemodynamic burden.

The observed associations between pleural effusion burden and markers of right-sided dysfunction are also of interest. Previous studies have demonstrated that pulmonary hypertension and impaired right ventricular–pulmonary arterial coupling are powerful indicators of disease severity and adverse cardiovascular remodeling in patients with severe AS undergoing TAVI [25,26]. Our findings extend these observations by suggesting that pleural effusion volume may represent an imaging correlate of these hemodynamic abnormalities. Nevertheless, these cardiovascular associations need to be interpreted within the broader clinical context of an elderly and multimorbid TAVI population. Pleural effusion should not be considered specific to cardiovascular congestion. Pleural fluid accumulation may also result from chronic pulmonary disease, malignancy, renal dysfunction, inflammatory conditions, hypoalbuminemia, and other systemic disorders. In our cohort, COPD and a history of malignancy were not associated with increasing pleural effusion burden, whereas renal dysfunction showed a modest association with pleural effusion volume. Nevertheless, the retrospective study design did not permit systematic adjudication of the underlying etiology of each pleural effusion, and other potential contributors, including inflammatory and nutritional factors, could not be comprehensively assessed. Accordingly, the observed associations with cardiovascular parameters should be interpreted as evidence that cardiovascular congestion and hemodynamic impairment may contribute to pleural effusion burden, rather than indicating that pleural effusion represents a specific marker of cardiac congestion.

The observed association with AF further supports this concept. AF is highly prevalent among elderly patients undergoing TAVI and is closely linked to elevated filling pressures, atrial myopathy, and advanced diastolic dysfunction [27,28]. Similarly, the correlation between pleural effusion volume and renal dysfunction likely reflects the well-established cardiorenal interactions that accompany chronic congestion and advanced cardiovascular disease [29]. Collectively, these observations suggest that pleural effusion volume integrates information from multiple interconnected pathophysiological domains and may therefore be viewed as a composite marker of cardiovascular disease burden rather than the consequence of a single underlying mechanism.

### 4.3. Why Does Pleural Effusion Not Predict Mortality After TAVI?

The absence of an independent association between AI-derived pleural effusion volume and long-term mortality requires consideration in light of recent TAVI studies reporting prognostic relevance of pleural effusions. Esin et al. [11] identified pre-procedural pleural effusion as an independent predictor of long-term mortality after TAVI, while Fischer-Rasokat et al. [12] demonstrated that a broader clinical fluid-overload phenotype, including rales, peripheral edema, and pleural effusion, was associated with increased one-year mortality. More recently, Järvinen et al. [13] reported that CT-detected pleural effusion, defined by a thickness greater than 10 mm, was independently associated with cardiovascular mortality in a large TAVI cohort. At first glance, these findings appear to contrast with our results.

However, important methodological and conceptual differences should be considered. Both Esin et al. [11] and Järvinen et al. [13] primarily evaluated pleural effusion as a binary or semi-quantitative finding, whereas the present study assessed pleural fluid burden across its entire measurable spectrum using AI-derived volumetric quantification. This distinction is clinically relevant. The presence of pleural effusion may identify a subgroup of patients with advanced disease, while volumetric assessment allows evaluation of whether increasing pleural fluid burden translates into progressively higher risk. In our cohort, no such dose–response relationship was observed. Neither continuous volume measurements, predefined thresholds of ≥100 mL and ≥500 mL, nor restricted cubic spline analyses demonstrated evidence of increasing mortality risk with increasing pleural effusion burden.

The findings reported by Fischer-Rasokat et al. [12] may be interpreted somewhat differently, as their prognostic signal was derived from a broader fluid-overload phenotype rather than from isolated pleural effusion itself. Such a phenotype likely captures multiple dimensions of clinical decompensation and systemic congestion that extend beyond the presence of pleural fluid alone. Consequently, the prognostic relevance observed in that study may reflect the overall burden of cardiovascular decompensation rather than the independent contribution of pleural effusion.

Viewed in this context, our findings may not contradict previous TAVI studies but rather refine their interpretation. Pleural effusion remains a clinically meaningful marker of advanced cardiovascular disease and congestion. However, the amount of pleural fluid itself does not appear to confer proportionally greater risk. The absence of a measurable dose–response relationship across categorical, continuous, threshold-based, and nonlinear analyses suggests that pleural effusion is better understood as a manifestation of cardiovascular vulnerability than as an independent prognostic phenotype after TAVI.

### 4.4. AI-Based Quantification and Clinical Implications

The present study also highlights the opportunities and limitations of AI-enabled opportunistic imaging [30]. Advances in automated image segmentation increasingly allow extraction of quantitative information from routinely acquired CT datasets without additional imaging, radiation exposure, or patient burden [31]. Pleural effusion volume represents a particularly suitable target for such approaches, as fluid accumulation can be quantified objectively and reproducibly across the entire spectrum of disease severity [14,15].

Importantly, however, the ability to quantify an imaging feature does not necessarily imply clinical or prognostic relevance. While AI-derived pleural effusion volume was associated with markers of cardiovascular dysfunction and congestion, it did not improve risk stratification for long-term mortality after TAVI. These findings emphasize that imaging biomarkers should not be evaluated solely on the basis of technical feasibility but rather according to the biological and clinical information they provide.

From a broader perspective, our results illustrate both the promise and the challenge of AI-driven imaging biomarkers. Automated quantification enables scalable and reproducible phenotyping of patients from routinely acquired CT datasets, potentially uncovering clinically meaningful features that would otherwise remain underutilized. At the same time, the present findings demonstrate that not every measurable imaging characteristic translates into incremental prognostic value. Future applications of AI in cardiovascular imaging may therefore benefit less from identifying isolated imaging findings and more from integrating multiple complementary markers into comprehensive phenotypic and risk assessment models.

## 5. Limitations and Strengths

The findings of the present study should be interpreted within the context of several limitations. Owing to its retrospective single-center design, residual confounding cannot be completely excluded despite comprehensive statistical adjustment. Furthermore, pleural effusion volume was assessed exclusively on pre-procedural CT imaging, precluding evaluation of temporal changes following TAVI. As relief of valvular obstruction may substantially influence congestion status and pleural fluid accumulation, longitudinal assessment could provide additional insights into the dynamic relationship between pleural effusions and clinical outcomes.

Furthermore, natriuretic peptide measurements were not systematically available in this retrospective cohort. Consequently, the relationship between pleural effusion volume and BNP or NT-proBNP could not be assessed, limiting our ability to directly relate AI-derived pleural fluid burden to a circulating biomarker of systemic congestion.

Although AI-based segmentation enabled objective and reproducible volumetric quantification, automated image analysis remains dependent on image quality and segmentation performance. To reduce the likelihood of measurement error, patients with insufficient image quality or inadequate segmentation quality were excluded prior to analysis. In addition, the present investigation focused on all-cause mortality as the primary endpoint. Whether pleural effusion burden may be associated with other clinically relevant outcomes, including heart failure hospitalization, symptom burden, or functional recovery after TAVI, remains uncertain.

Finally, the etiology of pleural effusion was not systematically adjudicated. Although relevant comorbidities such as COPD, malignancy, and renal dysfunction were captured, other potential causes of pleural fluid accumulation, including inflammatory disorders, hypoalbuminemia, and systemic illness, were not comprehensively available. Residual confounding by these factors therefore cannot be excluded, and pleural effusion volume should not be interpreted as a specific marker of cardiovascular congestion.

These limitations should be balanced against several important strengths of the study, including the use of a contemporary and comprehensively phenotyped TAVI cohort, long-term follow-up, detailed echocardiographic characterization, and AI-derived volumetric quantification of pleural effusion burden from routinely acquired CT datasets.

## 6. Conclusions

Pleural effusion is present in approximately one-quarter of patients undergoing TAVI and is associated with a broad spectrum of cardiovascular dysfunction characterized by pulmonary hypertension, right ventricular impairment, AF, and reduced cardiac performance. While AI-based quantification enables precise assessment of pleural effusion burden from routine CT datasets, pleural effusion volume does not provide independent prognostic information for long-term mortality after TAVI. Rather than representing a prognostic phenotype or a specific marker of cardiovascular congestion, pleural effusion appears to reflect a multifactorial imaging phenotype associated with cardiovascular and extracardiac disease burden in patients with advanced AS.

## Figures and Tables

**Table 1:** Distribution of AI-Derived Pleural Effusion Volume.

Group	Definition	*n* (%)
No effusion	0 mL	346 (73.62)
Small effusion	>0 to <100 mL	73 (15.53)
Moderate effusion	100 to <500 mL	26 (5.53)
Larger effusion	≥500 mL	25 (5.32)

**Table 2:** Baseline Characteristics According to Pleural Effusion Group.

Variable	Overall	No Effusion	Small Effusion	Moderate Effusion	Larger Effusion	*p*-Value
Demographics						
Age, years	82 [79–86]	82 [79–85]	82 [79–87]	83.5 [81.3–86.0]	82 [78–87]	0.423
Male sex, n (%)	239 (50.9)	165 (47.7)	40 (54.8)	17 (65.4)	17 (68.0)	0.073
BMI, kg/m^2^	25.2 [22.7–28.2]	25.3 [23.0–28.7]	24.4 [21.6–27.0]	24.7 [22.6–28.1]	24.1 [21.6–26.6]	0.086
BSA, m^2^	1.82 [1.68–1.95]	1.82 [1.67–1.94]	1.81 [1.69–1.95]	1.85 [1.70–2.00]	1.89 [1.77–1.95]	0.538
Clinical Characteristics						
NYHA ≥ III, n (%)	153 (38.1)	111 (37.8)	22 (34.4)	10 (45.5)	10 (45.5)	0.705
Diabetes mellitus, n (%)	122 (26.0)	88 (25.4)	21 (28.8)	10 (38.5)	3 (12.0)	0.172
AF, n (%)	175 (37.2)	111 (32.1)	34 (46.6)	13 (50.0)	17 (68.0)	<0.001
COPD, n (%)	52 (11.1)	34 (9.8)	10 (13.7)	5 (19.2)	3 (12.0)	0.417
Prior stroke, n (%)	39 (8.3)	30 (8.7)	6 (8.2)	2 (7.7)	1 (4.0)	0.877
History of malignancy, n (%)	77 (16.4)	52 (15.0)	13 (17.8)	6 (23.1)	6 (24.0)	0.479
Laboratory Parameters						
Hemoglobin, g/dL	12.9 [11.8–14.0]	13.0 [11.9–14.0]	12.6 [11.5–13.8]	13.4 [11.6–14.2]	11.8 [11.1–13.0]	0.028
Creatinine, mg/dL	1.00 [0.83–1.30]	0.98 [0.81–1.22]	1.21 [0.87–1.48]	1.12 [0.92–1.35]	1.20 [1.10–1.40]	0.001
Echocardiographic Parameters						
LVEF, %	55 [50–60]	55 [52–60]	55 [45–58]	45 [40–55]	55 [34.5–60]	<0.001
Mean AV gradient, mmHg	44.3 [39–50]	45 [40–51]	42 [37–50]	42.5 [35–47.8]	42.5 [36.5–50.4]	0.224
sPAP, mmHg	37 [27–48.5]	35 [26–45]	40 [27–52]	52.5 [36.5–59.5]	49 [41–65]	<0.001
TAPSE, mm	21.9 [19–25]	22 [19–25]	20 [18–23]	19 [18–20.8]	18 [14–23]	<0.001
TAPSE/sPAP	0.60 [0.42–0.87]	0.63 [0.48–0.92]	0.49 [0.36–0.77]	0.38 [0.30–0.51]	0.39 [0.27–0.56]	<0.001
Stroke volume index, mL/m^2^	43.7 [35.3–55.7]	46.2 [37.8–56.8]	40.5 [31.8–49.9]	36.2 [30.8–43.7]	34.8 [29.5–47.9]	<0.001

**Table 3:** Correlations Between Pleural Effusion Volume and Baseline Characteristics.

Variable	Spearman Rho	*p*-Value
Demographics		
Age, years	0.079	0.088
Male sex	0.116	0.012
BMI, kg/m^2^	−0.109	0.019
BSA, m^2^	0.054	0.245
Clinical Characteristics		
NYHA ≥ III	0.017	0.727
Diabetes mellitus	0.010	0.836
AF	0.189	<0.001
COPD	0.066	0.153
Prior stroke	−0.029	0.535
History of malignancy	0.059	0.200
Laboratory Parameters		
Hemoglobin, g/dL	−0.095	0.040
Creatinine, mg/dL	0.186	<0.001
Echocardiographic Parameters		
LVEF, %	−0.221	<0.001
Mean AV gradient, mmHg	−0.098	0.035
sPAP, mmHg	0.224	<0.001
TAPSE, mm	−0.236	<0.001
TAPSE/sPAP	−0.267	<0.001
Stroke volume index, mL/m^2^	−0.229	<0.001

**Table 4:** Univariate Cox Regression Analysis of Pleural Effusion and Long-Term Mortality.

Variable	HR (95% CI)	*p*-Value
Pleural effusion volume present	1.25 (0.91–1.72)	0.160
Pleural effusion volume per 100 mL	1.02 (0.97–1.07)	0.482
Log-transformed pleural effusion volume	1.02 (0.95–1.10)	0.515
Pleural effusion volume ≥ 100 mL	0.96 (0.60–1.54)	0.858
Pleural effusion volume ≥ 500 mL	0.93 (0.47–1.81)	0.822

Abbreviations: CI = confidence interval; HR = hazard ratio.

**Table 5:** Multivariable Cox Regression Analysis of Pleural Effusion and Long-Term Mortality.

Variable	Adjusted HR (95% CI)	*p*-Value
Model 1 *		
Pleural effusion volume present	1.05 (0.76–1.46)	0.761
Pleural effusion volume per 100 mL	0.99 (0.94–1.05)	0.849
Log-transformed pleural effusion volume	0.98 (0.91–1.05)	0.594
Pleural effusion volume ≥ 100 mL	0.77 (0.47–1.25)	0.290
Pleural effusion volume ≥ 500 mL	0.71 (0.36–1.41)	0.330
Model 2 **		
Pleural effusion volume present	1.24 (0.86–1.77)	0.245
Pleural effusion volume per 100 mL	1.01 (0.95–1.08)	0.661
Log-transformed pleural effusion volume	1.00 (0.93–1.08)	0.976
Pleural effusion volume ≥ 100 mL	0.79 (0.47–1.33)	0.383
Pleural effusion volume ≥ 500 mL	0.81 (0.40–1.61)	0.540

Model definitions: * Model 1 was adjusted for age, male sex, AF, and creatinine. ** Model 2 was adjusted for age, male sex, AF, creatinine, sPAP, TAPSE, LVEF, and stroke volume index. Abbreviations: AF = atrial fibrillation; CI = confidence interval; HR = hazard ratio; LVEF = left ventricular ejection fraction; sPAP = systolic pulmonary artery pressure; SVi = stroke volume index; TAPSE = tricuspid annular plane systolic excursion.

## Data Availability

The data presented in this study are available from the corresponding author upon reasonable request, subject to institutional and ethical restrictions. The data are not publicly available due to patient privacy and confidentiality considerations and restrictions imposed by institutional and ethical regulations governing the use of clinical patient data.

## Supplementary Materials

The following supporting information can be downloaded at: https://www.mdpi.com/article/10.3390/medsci14040472/s1, Supplementary Table S1: Restricted Cubic Spline Analyses of Log-Transformed Pleural Effusion Volume and Long-Term Mortality.

## Abbreviations

The following abbreviations are used in this manuscript: AF, atrial fibrillation; AI, artificial intelligence; AS, aortic stenosis; CT, computed tomography; CTA, computed tomography angiography; EACVI, European Association of Cardiovascular Imaging; HR, hazard ratio; IQR, interquartile range; LVEF, left ventricular ejection fraction; PACS, picture archiving and communication system; sPAP, systolic pulmonary artery pressure; SVi, stroke volume index; TAPSE, tricuspid annular plane systolic excursion; TAVI, transcatheter aortic valve implantation.
