# Supplementary material for: More than Fluid: AI-Derived Pleural Effusion Volume as a Marker of Cardiovascular Congestion in Transcatheter Aortic Valve Implantation
Source: Med Sci (Basel). 2026 Aug 11;14(4):472. doi: 10.3390/medsci14040472 (PMC13515112; doi:10.3390/medsci14040472)
Supplement: Supplementary file 1 [file medsci-14-00472-s001.zip › medsci-4463928-supplementary.pdf]

**Supplementary Table S1. Restricted Cubic Spline Analyses of Log-Transformed Pleural Effusion Volume and Long-Term Mortality**

| Variable | Wald $\chi^2$ | df | p-value |
|---|---|---|---|
| **Unadjusted model** | | | |
| Pleural log volume | 5.33 | 3 | 0.150 |
| Nonlinear component | 4.23 | 2 | 0.121 |
| Total | 5.33 | 3 | 0.150 |
| **Adjusted Model 1*** | | | |
| Pleural log volume | 2.42 | 3 | 0.490 |
| Nonlinear component | 2.41 | 2 | 0.300 |
| Age | 7.81 | 1 | 0.005 |
| Male sex | 7.12 | 1 | 0.008 |
| Atrial fibrillation | 5.38 | 1 | 0.020 |
| Creatinine | 0.46 | 1 | 0.496 |
| Total | 26.44 | 7 | <0.001 |
| **Adjusted Model 2**** | | | |
| Pleural log volume | 2.43 | 3 | 0.488 |
| Nonlinear component | 2.42 | 2 | 0.299 |
| Age | 7.92 | 1 | 0.005 |
| Male sex | 7.03 | 1 | 0.008 |
| Atrial fibrillation | 4.74 | 1 | 0.029 |
| Creatinine | 0.40 | 1 | 0.529 |
| sPAP | 0.19 | 1 | 0.667 |
| TAPSE | 0.22 | 1 | 0.642 |
| LVEF | 0.06 | 1 | 0.804 |
| SVi | 0.06 | 1 | 0.801 |
| Total | 26.85 | 11 | 0.005 |

Model definitions:

*Adjusted Model 1 was adjusted for age, male sex, atrial fibrillation, and creatinine.

** Adjusted Model 2 was adjusted for age, male sex, atrial fibrillation, creatinine, sPAP, TAPSE, LVEF, and stroke volume index.

Abbreviations:

LVEF = left ventricular ejection fraction; sPAP = systolic pulmonary artery pressure; SVi = stroke volume index; TAPSE = tricuspid annular plane systolic excursion.
